# DYNAMIC STANDING BALANCE AND MOBILITY IMPROVES WITHIN SIX MONTHS OF EXERCISE PARTICIPATION IN OLDER VETERANS

**DOI:** 10.1093/geroni/igae098.3954

**Published:** 2024-12-31

**Authors:** Kathleen Dondero, Jamie Giffuni, Megan Kelly, Odessa Addison

**Affiliations:** Towson University, Towson, Maryland, United States; Baltimore and Loch Raven VAMC, Baltimore, Maryland, United States; Baltimore Veterans Affairs Medical Center, Baltimore, Maryland, United States; University of Maryland School of Medicine, Baltimore, Maryland, United States

## Abstract

Older veterans are particularly vulnerable to declines in balance and mobility. Gerofit is a nationwide clinical exercise program aimed at improving physical function. However, it is unclear if Gerofit has an impact on balance, particularly in those that are already at high risk for falls. The Four-Square Step Test (FSST) is a valid and reliable measure of dynamic balance where completion times >12 seconds indicates a high risk for falls. The FSST was assessed at baseline, 6-months, and 1-year in 140 older Veterans who participated in the Gerofit exercise program. This sample included 88 “low fall risk” veterans (FSST < 12 seconds; age 71 ± 5.8 yrs; SPPB Score 11.08 ± 1.4; gait speed 1.2 ± 0.22 m/s) and 52 “high fall risk” veterans (FSST > 12 seconds; age 74 ± 8.1 yrs; SPPB Score 8.65 ± 2.2; gait speed 0.95 ±0.24 m/s). On average, FSST times improved ~13% from baseline (13.31 ± 7.6 seconds) to 6-months (11.55 ± 7.1seconds; P< 0.001) and was retained through 1 year of exercise participation (11.54 ± 7.1 seconds; P=1.0). Importantly, high fall risk veterans showed clinically meaningful improvements in FSST times from baseline (19.1 ± 1.4) to 6 months (15.2 ± 1.4; P=.001) in the program and maintained these improvements through 1-year (15.8 ± 1.4; P=1.0). Veterans, regardless of fall risk, can experience improvements in balance and mobility within 6 months of regular exercise participation and retain these improvements with continued exercise participation.

